# Heavy smoking during pregnancy as a marker for other risk factors of adverse birth outcomes: a population-based study in British Columbia, Canada

**DOI:** 10.1186/1471-2458-12-102

**Published:** 2012-02-06

**Authors:** Anders C Erickson, Laura T Arbour

**Affiliations:** 1 Division of Medical Sciences, University of Victoria, Medical Science Bld. Rm-104, STN CSC, PO Box 1700, Victoria, B.C., V8W 2Y2, Canada; 2 Department of Medical Genetics, University of British Columbia, Vancouver, British Columbia, Canada

## Abstract

**Background:**

Smoking during pregnancy is associated with known adverse perinatal and obstetrical outcomes as well as with socio-economic, demographic and other behavioural risk factors that independently influence outcomes. Using a large population-based perinatal registry, we assess the quantity of cigarettes smoked for the magnitude of adverse birth outcomes and also the association of other socio-economic and behavioural risk factors documented within the registry that influence pregnancy outcomes. Our goal was to determine whether number of cigarettes smoked could identify those in greatest need for comprehensive intervention programs to improve outcomes.

**Methods:**

Our population-based retrospective study of singleton births from 2001 to 2006 (N = 237,470) utilized data obtained from the BC Perinatal Database Registry. Smoking data, self reported at the earliest prenatal visit, was categorized as: never, former, light (1 to 4), moderate (5 to 9), or heavy smoker (10 or more per day). Crude and adjusted odds ratios (AOR) with 95% confidence intervals (95% CI) were calculated using logistic regression models for smoking frequency and adverse birth outcomes. A partial proportional odds (pp-odds) model was used to determine the association between smoking status and other risk factors.

**Results:**

There were 233,891 singleton births with available smoking status data. A significant dose-dependent increase in risk was observed for the adverse birth outcomes small-for-gestational age, term low birth weight and intra-uterine growth restriction. Results from the pp-odds model indicate heavy smokers were more likely to have not graduated high school: AOR (95% CI) = 3.80 (3.41-4.25); be a single parent: 2.27 (2.14-2.42); have indication of drug or alcohol use: 7.65 (6.99-8.39) and 2.20 (1.88-2.59) respectively, attend fewer than 4 prenatal care visits: 1.39 (1.23-1.58), and be multiparous: 1.59 (1.51-1.68) compared to light, moderate and non-smokers combined.

**Conclusion:**

Our data suggests that self reports of heavy smoking early in pregnancy could be used as a marker for lifestyle risk factors that in combination with smoking influence birth outcomes. This information may be used for planning targeted intervention programs for not only smoking cessation, but potentially other support services such as nutrition and healthy pregnancy education.

## Background

Smoking during pregnancy is associated with known adverse perinatal and obstetrical outcomes [1-4]; however, it remains unclear the magnitude as to which adverse outcomes are related to cigarette smoke itself versus surrounding factors difficult to quantify and control. For instance, socio-economic status (SES) and psychosocial stress are both associated with adverse birth outcomes [5-7] as well as with prevalence of smoking during pregnancy [8-10]. These observations are supported by the mounting biological evidence for a stress-related psychoneuroendocrine process contributing to the underlying etiology of adverse fetal development [11]. The linkages between socially patterned adverse health behaviours and outcomes are difficult to understand let alone separate and measure. Therefore, it may be beneficial to use (heavy) smoking during pregnancy as a marker for latent and unquantifiable risk factors that also affect outcomes.

In a recent report assessing the number of cigarettes smoked during pregnancy and adverse birth outcomes in the Qikiqtaaluk (Baffin) region of Nunavut, 'heavy smokers' (greater than ten cigarettes per day) had significantly worse birth outcomes than non- and light smokers [12]. In the Qikiqtaaluk population where 80% of pregnant mothers smoke, it was surprising to observe what resembles a threshold effect of heavy smoking on adverse birth outcomes, particularly birth weight. A dose-response relationship was also observed between level of smoking during pregnancy and higher self-reporting of alcohol or drug use (predominately marijuana). Despite certain limitations, the results led to the conclusion that heavy smoking may be a marker for additional risk factors and be used to identify high risk populations for targeted intervention [12].

In addition to the inter-relationship between adverse birth outcomes, SES and psychosocial stress mentioned above, heavy smoking could also be marker for poor nutritional status [13]. Smokers in general are shown to have poorer nutritional profiles than non-smokers in which behavioural and biological factors independently account for the differences, particularly micronutrients, essential minerals and vitamins [14,15]. While smokers tended to have reduced dietary intake of some micronutrients, the observed lower blood/serum concentrations were primarily attributed to the increased turnover of micronutrients via an inflammatory response caused by the oxidative stress of smoking. Further, in certain cases the inflammation ascribed effect was more pronounced in long-term and heavy smokers [14]. The effects are further confounded amongst pregnant women where it has been shown that heavy smoking, low social class, renting accommodation, and low education predict poor dietary intake [16].

As important as understanding the etiology of adverse birth outcomes, is identifying those at particular risk who might benefit from intervention with the goal of improving outcomes at the population level. The purpose of this study is two-fold: 1) to assess the quantity of cigarettes smoked and the magnitude of adverse birth outcomes, and 2) determine the association of quantity of cigarettes smoked with other socio-economic and behavioural risk factors documented within the registry that also influence pregnancy outcomes. We used a well-established perinatal registry database to ask the question: can high quantities of cigarette use reported at the first prenatal visit be used as a surrogate to identify high risk mothers for targeted support services throughout pregnancy?

## Methods

This population-based retrospective study of all singleton births (live born and stillbirths) in British Columbia from 2001 to 2006 (N = 237,470) utilized the Perinatal Services British Columbia (PSBC) Perinatal Database Registry, and included information on maternal-infant health status and outcomes, reproductive history, socio-demographics and residential postal codes. The PSBC accounts for 99% of about 45,000 births and stillbirths per year occurring in the province from 20 weeks gestation or weighing at least 500 g at birth or stillbirth. Third party data access is provided by a Partnership Accord/Memorandum of Agreement between all B.C. Health Authorities and the PSBC through the *Freedom of Information and Privacy Protection Act *[17]. Research ethics board approval was granted by the Research Review Committee at PSBC and the University of Victoria.

The outcome variables included low birth weight at term (LBW < 2,500 g with ≥ 37 weeks gestation), preterm birth (PTB-between 20 and 36 completed weeks gestation), intra-uterine growth restriction (IUGR -identified during the antenatal period using ultrasound imaging growth parameters), postnatal small-for-gestational age below the third and tenth percentiles for weight and sex using BC specific birth charts (SGA-3 and SGA-10 respectively) [18], and stillbirths (≥ 20 weeks gestation or ≥ 500 g). Out-of-province (n = 926), records missing geographic data on maternal area of residence (n = 129), and records not meeting the criteria of a recorded birth in BC (< 20 weeks gestation and < 500 g birth weight, n = 12) were excluded. Outcomes were reviewed for completeness and checked for double counting between variables (e.g. stillbirth and SGA).

Smoking data is usually collected at the first prenatal visit from 12 to 18 weeks gestation and is categorized in the Registry as "never", "former", and "current". The number of cigarettes smoked per day by current smokers was available as an additional continuous variable and was categorized into three levels of daily maternal cigarette use: light (1 to 4), moderate (5 to 9), and heavy (10 or more). In terms of former smokers, it is unknown when in relation to the pregnancy cessation took place prior to the first prenatal visit. Despite being non-smokers, former smokers were not combined with the 'never' smoked group due to significant maternal characteristic differences between them. The additional individual-level variables include: maternal age, reproductive history (parity ≥ 1), number of antenatal care visits, co-morbidities such as diabetes, gestational diabetes and hypertension during pregnancy, pre-pregnancy weight, indication of drug or alcohol use, number of school years completed and single parent status (indication of support during and after the pregnancy).

Bivariate odds ratio (OR) tests and 95% confidence intervals (95% CI) were calculated to assess the influence of each covariate on birth outcomes with the results informing which covariates to include in the models. Crude and adjusted ORs with 95% CIs were calculated using logistic regression to assess the influence of smoking rates on outcomes. Sensitivity analyses were conducted to assess the influence of attrition due to missing data for some covariates. This included bivariate OR tests to determine the likelihood of adverse birth outcomes and maternal characteristics between records with and without data.

In order to determine the association between the covariate risk factors and the different levels of maternal smoking, a specialized case of an ordered logistic model was used called the *partial proportional odds (pp-odds) model*. An ordered (ordinal rank) logistic model is equivalent to a series of binary logistic regressions where the different levels or group ranks of the dependent variable are combined and contrasted [19]. In this case, there are four ordinal levels of smoking status (Never, Light, Medium and Heavy) where: Level 1 is contrasted with Levels 2,3, and 4 combined; Levels 1 and 2 combined versus Levels 3 and 4 combined; and Levels 1,2, and 3 combined versus Level 4. The pp-odds model is less restrictive compared to a regular ordered logistic model (also known as a parallel-lines or proportional-odds model), which assumes all β regression coefficients to be parallel. The pp-odds model eases this restriction allowing some β coefficients to be the same and some to differ [19]. Former smokers were not used in this analysis due to its non-ordinal status. Interactions between covariates were checked with no significant interaction effects observed. All analyses were conducted in *Stata 11 IC *[20].

## Results

**Figure 1:**
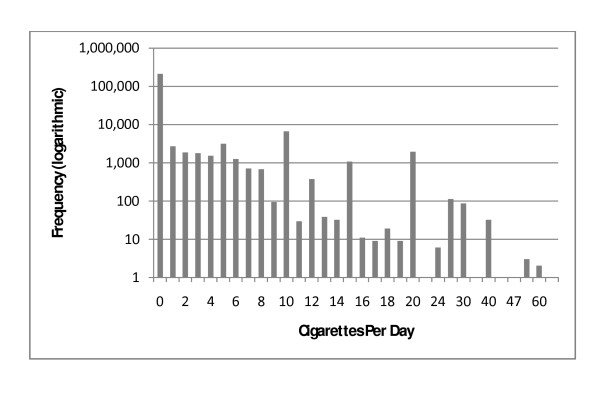
**Distribution of Maternal Daily Cigarette Consumption in BC, 2001-06**.

Between 2001 and 2006, there were 236,403 singleton births ≥ 500 g or over 20 weeks gestation in BC. Among them, 26,564 (11.2%) were active smokers, 197,583 (83.6%) reported never smoking, and 12,256 (5.2%) were former smokers. Of the active smokers, 7,806 (3.3% of total *N*) were light smokers (1-4 cigarettes/day), 5,839 (2.5%) were moderate smokers (5-9 cigarettes/day), 10,407 (4.4%) were heavy smokers (≥ 10 cigarettes/day), and 2,512 (1.1%) had missing data on the number of cigarettes smoked per day which were excluded from the analysis. A comparison of the maternal characteristics across smoking groups is provided in Table 1. The distribution of daily cigarette consumption was exponential with notable spikes at increments of five cigarettes per day (Figure 1).

**Table 1:** Maternal characteristics by smoking status in BC, 2001-2006

		Births by smoking status,* %						
		**Never**	**Former**	**1-4**	**5-9**	**10 +**	**Missing**	**Total (% missing)**
**Characteristic**		***n = 197,583***	***n = 12,256***	***n = 7,806***	***n = 5,839***	***n = 10,407***	***n = 2,512***	***n = 236,403 (1.1)***

Maternal Age	< 20	2.3	7.8	14.0	12.2	9.2	11.8	8,620 *(3.5)*
	
	20-24	12.4	24.3	32.4	32.7	30.3	29.0	35,817 *(2.0)*
	
	25-29	27.9	29.5	26.8	27.2	26.8	25.7	65,877 *(1.0)*
	
	30-34	34.7	24.5	17.4	17.3	20.3	21.1	76,616 *(0.7)*
	
	35-39	18.7	11.5	7.7	8.8	10.8	10.1	40,808 *(0.6)*
	
	40+	4.0	2.4	1.7	1.8	2.5	2.2	8,665 *(0.7)*

Parity ≥ 1	No	44.3	59.3	54.7	47.3	40.2	52.5	107,381 *(1.2)*
	
	Yes	55.7	40.7	45.3	52.7	59.8	47.5	129,022 *(0.9)*

Single Parent	No	92.7	87.1	76.5	77.8	75.1	75.6	214,054 *(0.9)*
	
	Yes	3.7	8.7	15.0	15.1	16.7	18.3	12,726 *(3.6)*
	
	Unknown	3.6	4.1	8.6	7.1	8.2	6.1	9,623 *(1.6)*

Has Grade 12†	No	0.8	3.0	5.2	5.2	5.4	3.1	3,301 *(0.7)*
	
	Yes	10.7	12.9	9.6	9.4	6.5	6.7	24,908 *(2.4)*
	
	missing	88.5	84.1	85.2	85.4	88.1	90.2	208,194 *(1.1)*

Gestational	No	93.3	94.6	96.2	95.8	95.1	94.7	221,256 *(1.1)*
	
diabetes	Yes	6.7	5.4	3.8	4.2	4.9	5.3	15,147 *(0.9)*

Pre-existing Diabetes	No	99.6	99.7	99.6	99.6	99.4	99.6	235,465 *(1.1)*
	
	Yes	0.4	0.3	0.4	0.4	0.6	0.4	938 *(1.2)*

Hypertension in pregnancy	No	97.8	97.1	98.1	98.0	98.2	97.8	228,674 *(1.1)*
	
	Yes	2.2	3.0	1.9	2.0	1.8	2.2	5,272 *(1.0)*

Indication of Alcohol Use	No	99.7	98.1	95.9	96.6	95.1	93.8	234,290 *(1.0)*
	
	Yes	0.4	1.9	4.1	3.4	4.9	6.3	2,113 *(7.4)*

Indication of Drug Use	No	99.2	96.9	90.8	90.3	84.6	86.7	231,267 *(0.9)*
	
	Yes	0.8	3.1	9.2	9.7	15.4	13.1	5,136 *(6.4)*

Pre-pregnancy weight	< 55	21.0	16.4	19.0	19.9	18.8	16.4	48,430 *(0.9)*
	
	55-74	42.9	43.6	38.8	38.6	36.2	35.0	99,973 *(0.9)*
	
	> 74	14.7	22.0	18.1	18.4	20.3	16.7	36,720 *(1.1)*
	
	missing	21.5	18.1	24.1	23.1	24.7	31.8	51,280 *(1.6)*

Prenatal Care Visits	≥ 3	92.1	93.4	92.3	91.2	89.5	85.9	217,461 *(1.0)*
	
	< 3	1.2	1.2	2.5	3.0	3.4	3.8	3,364 *(2.9)*
	
	missing	6.7	5.5	5.2	5.8	7.1	10.3	15,578 *(1.7)*

* Likelihood-Ratio Chi-squared tests for independence across the 5 smoking categories were all significant at *p *< 0.05, except pre-existing diabetes (*p = 0.06*).

† Maternal education was measured in years of education with '12 years' indicating high school education.

Maternal characteristics varied substantially across levels of maternal smoking (Table 1). Mothers who were heavy smokers were more likely to be multiparous, a single parent, had not completed high school, be identified for alcohol or drug use and attended fewer prenatal care visits. Heavy smokers were less likely to have had hypertension during the pregnancy and all smokers were less likely to have gestational diabetes. The highest proportion of smokers was under 25 years of age, but tended to be light smokers (inter-quartile range, IQR = 3-6-10). In contrast, the smaller proportion of older mothers (≥ 35) who did smoke tended to be heavy smokers (IQR = 4-10-10). Within age cohorts, a third of all mothers less than 20 years of age and nearly a quarter of women aged 20 to 24 reported smoking with 11.5 and 9% of those being heavy smokers respectively. Conversely, ten percent of the oldest three age cohorts report smoking but had roughly twice the proportion of heavy smokers as light smokers (3.6 and 1.6% respectively). Consistent with the youngest group reporting the greatest proportion of current smokers, the two youngest age cohorts also had the highest proportion of former smokers, 13.1 and 8.5% respectively.

Further bivariate OR tests with maternal age revealed that women under the age of 30, but particularly teens (under 20) and those 20 to 24 were significantly more likely to be identified for drug use, OR (95% CI) = 9.06 (8.19-10.03) and 4.36 (4.00-4.74) respectively; alcohol use, OR (95% CI) = 9.44 (8.13-10.97) and 3.87 (3.40-4.42) respectively; and attend fewer than 4 prenatal care visits, OR (95% CI) = 4.45 (3.91-5.07) and 2.35 (2.12-2.60) respectively compared to women 30 to 34 years of age. Lack of high school graduation for women aged 20 to 24 were also low compared to the 30-34 age cohort, OR (95% CI) = 9.80 (8.60-11.39). Furthermore, among women under 25 years of age, those who were heavy smokers were over ten times more likely be identified for drug use than non-smokers whereas light and moderate smokers had about half the risk, OR (95% CI) = 10.59 (9.46-11.86) and 6.67 (5.79-7.67) for heavy and moderate smokers respectively. While heavy smokers under 25 years old also had the highest risk for alcohol indication, low prenatal care attendance, single parent and no grade 12 educations, the differences between levels of smoking were less stark.

Missing values were a concern in two key covariates, education level and pre-pregnancy weight, therefore sensitivity analyses were carried out to determine the difference in characteristics of those with missing data. Sensitivity analyses on those missing education data (88.1%) revealed small but significantly increased risks for most adverse birth outcomes (OR range between 1.11 and 1.29) and no statistical difference for stillbirths. Light, moderate and former smokers were less likely to be missing education data compared to those who never smoked, while there was no difference between heavy smokers and never smokers with missing education data. However, overall differences in age and cigarettes consumption were negligible with the IQR of those with and without data being 3-6-10 and 3-7-10 respectively for cigarettes use among smokers and an identical IQR for age. The sensitivity analyses on those records missing pre-pregnancy weight data (21.7%) demonstrated significantly increased risks of PTB and stillbirths, a significantly lower risk of IUGR, and no statistical difference for the other outcomes. All levels of smoking were significantly more likely to be missing pre-pregnancy weight data compared to mothers who never smoked, although the IQR of cigarette use between those with and without data were similar (3-6-10 and 3-7-10 respectively). In terms of other maternal characteristics, there were some differences in the variables which were more or less likely to be missing data, however no clear trends were observed.

**Figure 2:**
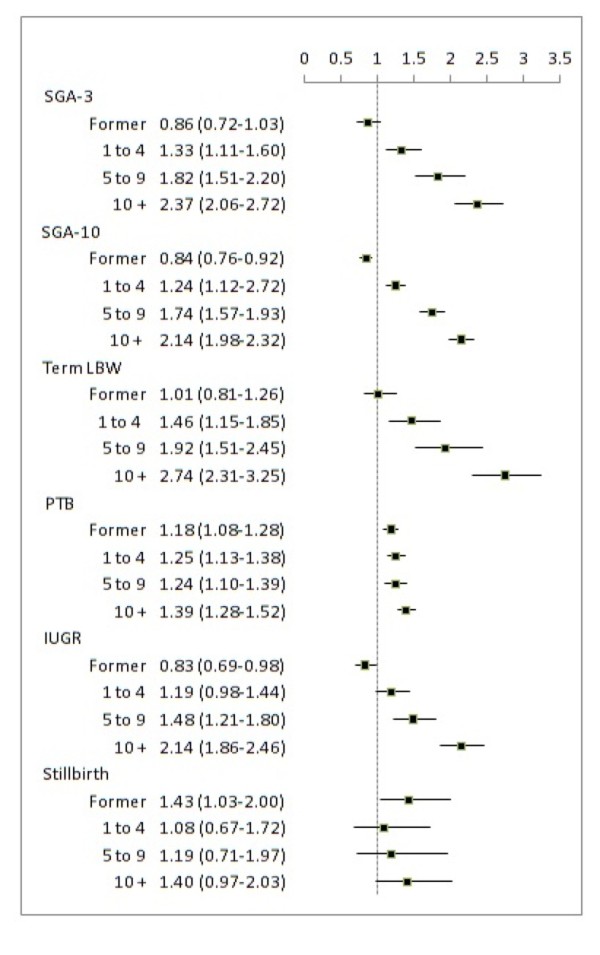
**Adjusted Odd Ratios of Adverse Birth Outcomes and Levels of Maternal Smoking**. SGA-3-Small for Gestational Age below the 3^rd ^percentile (*n *= 172,667), SGA-10-Small for Gestational Age below the 10^th ^percentile (*n *= 172,667), LBW-Low Birth Weight at term (*n *= 161,041), PTB-Preterm Birth (*n *= 172,690), IUGR-Intra-Uterine Growth Restriction (*n *= 172,849), Stillbirth (*n *= 173,397). Tests were adjusted for: maternal age, parity > 1, alcohol flag, drug flag, prenatal care visits, prior and gestational diabetes, hypertension during pregnancy, pre-pregnancy weight, and lone parent.

Figure 2 shows the adjusted odds ratios of adverse birth outcomes with maternal smoking status (differences between crude and adjusted ORs were unremarkable). Compared to mothers who never smoked, there was a significant dose-dependent increase in risk for all adverse birth outcomes with maternal smoking with the exception of stillbirths. Furthermore, heavy smokers had a significantly greater risk of SGA-3, SGA-10 and IUGR compared to light and moderate smokers. The addition of the education variable attenuated the effect of light smokers for all outcomes resulting in no significant difference compared to non-smokers. Similarly, all observed effects of smoking and PTB were reduced to the null after including the education variable to the model. However, the education variable did not alter the effect of outcomes on heavy smokers while it strengthened the effect of moderate smokers for SGA-3, SGA-10 and LBW.

Results from the pp-odds model describe how different covariate risk factors predict higher or lower levels of maternal smoking (Table 2). All variables except older maternal age were risk factors for smoking during pregnancy, but of those, only multiparity and pre-pregnancy weight greater than 74 kg predicted heavy smoking over the lower levels of smoking. This is demonstrated by the increasing effect of these two variables across the three comparisons. Older maternal age also predicted higher levels of smoking despite being associated with never smoking. Young maternal age (< 25 years), single parent, drug and alcohol indicators were all strongly associated with maternal smoking across all comparisons, but exhibited their strongest effects for Level-1 (never smoked versus all smokers). For example, women who smoked were 10 times more likely to be indicated for drug use compared to women who never smoked, while heavy smokers were 7.6 times more likely to be indicated for drug use compared to moderate, light and non-smokers combined. Having three or fewer prenatal care visits and a diagnosis of pre-existing diabetes (*p = 0.06*) met the parallel-lines assumption, and therefore had a constant effect across all levels of comparison. These general trends were sustained with the addition of the education variable into the pp-odds model demonstrating a strong constant effect across all levels of comparison, OR = 3.80 (95% CI 3.41-4.25) with a reduced population size of 21,775. Only the variables prenatal care and pre-existing diabetes had their effects significantly reduced to the null (*p = 0.9 *and *0.4 *respectively).

**Table 2:** Odds ratios of covariate risk factors predicting level of maternal smoking in B.C. 2001-2006 (n = 163,867)

Characteristic	OR (95% CI)	OR (95% CI)	OR (95% CI)
	
	**Level 1 Vs**.	Level 1+2	Level 1+2+3
	Level 2+3+4	Vs. Level 3+4	Vs. Level 4
**Young Maternal Age (< 25)**	3.66 (3.52-3.80)	3.27 (3.13-3.42)	2.86 (2.70-3.02)

**Older Maternal Age (≥ 35)**	0.67 (0.64-0.71)	0.71 (0.67-0.76)	0.77 (0.71-0.83)

**Single Parent**	2.42 (2.31-2.53)	2.25 (2.14-2.37)	2.27 (2.14-2.42)

**Parity ≥ 1**	1.26 (1.22-1.31)	1.49 (1.43-1.55)	1.59 (1.51-1.68)

**Alcohol Indication**	3.06 (2.63-3.57)	2.41 (2.08-2.81)	2.20 (1.88-2.59)

**Drug Indication**	10.19 (9.32-11.15)	8.17 (7.50-8.90)	7.65 (6.99-8.39)

**Prenatal Care Visits (≤ 3)**	1.39 (1.23-1.58)	1.39 (1.23-1.58)	1.39 (1.23-1.58)

**Pre-existing Diabetes**	1.27 (0.99-1.64)	1.27 (0.99-1.64)	1.27 (0.99-1.64)

**Pre-pregnancy weight (≥ 75 kg)**	1.48 (1.43-1.54)	1.49 (1.43-1.56)	1.56 (1.48-1.65)

Level 1 = never smoked, Level 2 = light smoker, Level 3 = moderate smoker, Level 4 = heavy smoker.

## Discussion

The results of this large population-based study support that smoking during pregnancy is a modifiable dose-dependent risk factor of adverse fetal growth that also has a strong relationship with other risk behaviour and low SES indicators. Compared to all lower levels of smoking, heavy smokers (≥ 10 cigarettes/day) had substantially worse birth outcomes and were also at increased risk to be identified for alcohol use and drug use, be a single parent, attended fewer prenatal care visits and have pre-pregnancy weight greater than 74 kg. Although the addition of a major SES variable, level of education, was limited to only 10% of our study population, the main effects and general trends were corroborated. Heavy smokers were 3.8 times more likely to have not graduated high school compared to moderate, light and non-smokers combined supporting the possibility that reports of smoking greater than ten cigarettes per day might be an early marker for the need for comprehensive supports to reduce adverse outcomes.

The adjusted ORs for the impaired fetal growth outcomes (SGA, IUGR and term-LBW) were nearly twice the magnitude between heavy and light smokers. The addition of the education variable into the logistic models attenuated the effect of light smokers to the degree of no significant difference between light, former and never smokers while the effect of moderate and heavy smoking remained relatively stable with roughly double the risk. The effect of smoking on PTB was completely removed after adjusting for maternal education. This suggests that while behavioural and SES indicator variables, particularly maternal education, explain some or all of the risk attributed to light smoking, heavy smoking remains a robust marker of increased risk for the impaired fetal growth outcomes. Whether this observed effect is strictly biological or is partially a marker for some latent unmeasured risk factor, heavy smoking readily identifies approximately 5% of the BC population who could benefit from additional support services. These results were consistent with findings from a population-based study from Nova Scotia [21] as well as a prospective cohort study that used anthropometric ultrasound measurements to compare fetal growth in smoking and non-smoking expectant mothers [22].

The mechanisms to which cigarette smoke exposure effects fetal growth is not completely understood; however, IUGR correlates with defects in placental transport and metabolism functions which seems to restrict nutrient supply [23]. Zdravkovic et al. report that constituents in cigarette smoke directly affect placental cytotrophoblast proliferation and differentiation which reduces blood flow and creates a hypoxic environment [24]. Using a mouse model, Detmar et al. found that polycyclic aromatic hydrocarbons (PAHs), a main component in cigarette smoke, caused IUGR in the fetuses of exposed dams and yielded alterations in placental vascularisation with significantly reduced arterial surface area and volume [25]. PAHs are also a main constituent of vehicular exhaust, particularly diesel, and there is mounting evidence of an association between said pollutant and growth restricted birth outcomes [26,27].

The results from the pp-odds model show that most of the covariate risk factors primarily predict maternal smoking in general versus non-smokers. Variables such as single parent, drug and alcohol indication and young maternal age were significant across all levels comparison, but had the strongest effects in comparing non-smokers to all other levels of smoking. Conversely, parity exhibited its strongest effects in the third comparison (heavy smokers versus never, light, and moderate smokers combined). This suggests that while being multiparous is a marker for maternal smoking in general, it predicts heavy smoking versus moderate or light smoking habits. A similar observation was found in a study of UK women regarding gravida and smoking behaviour in subsequent pregnancies, commenting on the double exposure of the previous children to cigarette smoke both pre- and postnatally [28]. While older maternal age was associated with having reported 'never smoked', older mothers who did smoke were more likely to be heavy smokers. This trend of older mothers being heavy smokers was also observed in the Nunavut chart review study [12].

The results for the pp-odds model including maternal education generally hold true to the first model. The effects for age, parity, single parent, drug flag, and alcohol flag are slightly attenuated but remain significant with the same trend. Education (no grade 12) had a strong constant effect on maternal smoking across all levels of comparison, suggesting an important role in health literacy. Maternal level of education has been shown to be a powerful determinant of perinatal health, independent of, and stronger than that of neighbourhood income [29]. Having low maternal educational attainment, being young and a single parent are indicators of low socio-economic status that may exert additional stress on the pregnancy. The biochemical response to stress via elevated basal cortisol levels has been associated with low birth weight [30]. Three major systems are thought to be involved in the biological pathway linking maternal mental health and stress with adverse birth outcomes which include the neuroendocrine, the immune/inflammatory, and the cardiovascular systems with placental corticotrophin releasing hormone playing a central coordinating role [31]. Indicators of women's mental health during pregnancy such as psychosocial stress, level of social and financial support and depression may be one possible pathway to which low SES is associated with adverse birth outcomes.

The majority of results from this British Columbia based study were consistent with recent findings from Norway [32], Germany [33], and a national Canadian survey that analyzed the associated risk factors of smoking during pregnancy [8]. The Canadian study found that non-immigrant, single parent, low household income, no/little prenatal classes, less education, passive (i.e. partner) smoking, older maternal age and a higher number of stressful events were significantly associated with maternal smoking in general but did not assess quantity of cigarettes smoked [8]. An Australian study of similar design to our research also used registry data and found young maternal age, lack of antenatal care and low SES were associated with maternal smoking [34]. Both papers highlighted the importance of antenatal care as a critical access point to educate expectant mothers regarding a healthy pregnancy. Importantly, the study from Australia found that first-time mothers and those who accessed prenatal care early in their pregnancies had an increased likelihood of smoking cessation [34].

The province of BC has a relatively healthy birthing population compared to the rest of Canada and has amongst the lowest rates of maternal smoking and exposure to 2^nd ^hand smoke in Canada [35]. Further, BC has high grade 12 completion rates among pregnant women which likely influence the relatively low rates of risk behaviours such as maternal smoking. BC had lower rates of preterm birth and is around the Canadian average for rates of SGA. Despite these positive outcomes, the ability to recognize those at particular risk early in pregnancy and provide preventative programs could help achieve better outcomes for all expectant mothers. Specifically, our findings suggest that heavy maternal smoking will identify approximately 5% of women in BC at particular increased risk of adverse outcomes that may benefit from additional services to promote a healthy pregnancy. With respect to epidemiological analysis of population-based perinatal datasets, there is potential to use heavy maternal smoking as a proxy for unreliable or unmeasured individual-level behavioural and/or socio-economic data. Maternal self-reported smoking tends to be routinely collected for most birth registries making it an accessible variable compared to many other risk factor variables or when linkage to external data in not available.

There were several limitations to this analysis. First, there were no data on passive smoking rates (i.e. exposure to environmental tobacco smoke or having a partner who smokes), psychosocial stress, ethnicity, whether the pregnancy was planned, birth intervals for multiparous women, potential occupational exposures, or household income. Further, some of the covariates examined had high missing data. As described earlier, pre-pregnancy weight was missing approximately 25% of its values, and maternal education data were only available for 28,210 records (12%). The greatest concern when an important variable is poorly populated, is that the absence/presence of values is biased (i.e. are not missing at random). For instance, care providers may only be asking those individuals about their education status where literacy is a concern, and as a result the distribution would be biased and shifted to the left. Therefore missing data not only reduces the statistical power due to list-wise deletion (i.e. records with missing data are not used in that particular test), but also reduces the overall reliability of that variable and potentially the appropriateness of the model.

To address this potential bias, sensitivity analyses were carried out for level of education and pre-pregnancy weight. These tests demonstrated that records with missing education data tended to have small increased risks for all adverse birth outcomes and some maternal risk characteristics; while those missing pre-pregnancy weight data had mixed birth outcomes results but increased risk for most maternal characteristics. However, the overall age structure and cigarette consumption between those with and without missing data were nearly identical with similar medians and inter-quartile ranges. Taken as a whole, these results suggest that the missing education and pre-weight data may result in underestimating the risk of some adverse birth outcomes but the degree of missing data is relatively consistent across the levels of smoking and therefore it would be predicted not to affect the observed general trends of our analyses. Further, a review of the mean number of years of education for the Canadian female population (age 25-36) fall within the mean and standard deviation of our maternal years of education variable, 14.2 versus 13.9 ± 2.6 [36]. However, given the strong association between maternal education, smoking and risk of adverse outcomes, these results reinforce the importance for the standardized and complete collection of SES variables for *all *patients by all prenatal health care providers.

Another potential limitation is the self-reporting bias of cigarette consumption. Self-reported smoking status among pregnant women is susceptible to bias, and may lead to attenuation of the true effect of smoking on birth outcomes [37]. Rates of misclassification in the United States using data collected from the National Health and Nutrition Examination Surveys (NHANES) estimated non-disclosure to be around 20% [38]. The rate of misclassification was consistent with other studies [39], as was the demographic predictors of non-disclosure. Many studies used serum, salivary, or urinary cotinine as a biomarker to assess the degree of non-disclosure in smoking status, and have found a range of between 13% and 25% depending on the cut-off values used to classify one as an active smoker. Non-disclosure was higher among those who reported they were former smokers, and younger maternal age (20-24). The stigma around maternal smoking may lead some respondents to under report their actual consumption habits. Possible recall bias can also be assumed given the peaks in the histogram (Figure 1) corresponding at multiples of 5. This could be due to responses given in terms of some fraction in packs of cigarettes per day, such that half a pack is equal to ten cigarettes. None the less, our results suggest that *reported *number of cigarettes smoked correlates with adverse birth outcomes and associated socio-economic risk factors suggesting the information as provided will help identify those at highest risk.

Future analyses may include running a hierarchical multilevel model with the inclusion of neighbourhood-level deprivation scores to determine if clustering of observations by neighbourhoods regarding birth outcomes, smoking rates or prenatal care attendance. This type of analysis would be useful as a baseline to further study the effect of local air pollution exposure measured at the neighbourhood-level on birth outcomes and the potential interactions with SES and other risk variables.

## Conclusion

We have demonstrated that self reports of heavy smoking (≥ 10 cigarettes/day) early in pregnancy could be used as a marker for latent or other often unmeasured lifestyle risk factors that influence birth outcomes. Heavy smokers had worse outcomes and were substantially more likely to demonstrate other risk factors compared to other levels of smoking. While strategies for smoking cessation are important and supported by our study, the underlying issues that lead to adverse birth outcomes might not be addressed with a narrow focus. This information may be used for planning targeted intervention programs not only for smoking cessation but potentially other maternal support services such as nutrition and healthy pregnancy education with the overall goal of optimizing birth outcomes.

## Competing interests

The authors declare that they have no competing interests.

## Authors' contributions

Both AE and LA made substantial contributions with regards to data acquisition, study conception, design and interpretation. AE carried out the database management, statistical analyses, drafting the manuscript and making revisions. LA provided critical edits of the intellectual content. All authors read and approved the final manuscript.

## Pre-publication history

The pre-publication history for this paper can be accessed here:

http://www.biomedcentral.com/1471-2458/12/102/prepub
